# Gynecologist Supply Deserts Across the VA and in the Community

**DOI:** 10.1007/s11606-022-07591-5

**Published:** 2022-08-30

**Authors:** Sarah Friedman, Jonathan G. Shaw, Alison B. Hamilton, Kavita Vinekar, Donna L. Washington, Kristin Mattocks, Elizabeth M. Yano, Ciaran S. Phibbs, Amanda M. Johnson, Fay Saechao, Eric Berg, Susan M. Frayne

**Affiliations:** 1 VA HSR&D Center for Innovation to Implementation (Ci2i), VA Palo Alto Health Care System, Palo Alto, CA USA; 2 School of Public Health, University of Nevada Reno, Reno, NV USA; 3 Stanford University School of Medicine, Stanford, CA USA; 4 VA HSR&D Center for the Study of Healthcare Innovation, Implementation & Policy, VA Greater Los Angeles Healthcare System, Los Angeles, CA USA; 5 David Geffen School of Medicine, University of California, Los Angeles, Los Angeles, CA USA; 6 VA Central Western Massachusetts Healthcare System, Leeds, MA USA; 7 University of Massachusetts Medical School, Worcester, MA USA; 8 Fielding School of Public Health, University of California, Los Angeles, Los Angeles, CA USA; 9 VA Health Economics Resource Center, Menlo Park, CA USA; 10 VA Office of Women’s Health, Washington, DC USA

**Keywords:** gynecology supply, access to care, women veterans

## Abstract

**Background:**

The Veterans Health Administration (VA) refers patients to community providers for specialty services not available on-site. However, community-level specialist shortages may impede access to care.

**Objective:**

Compare gynecologist supply in veterans’ county of residence versus at their VA site.

**Design:**

We identified women veteran VA patients from fiscal year (FY) 2017 administrative data and assessed availability of a VA gynecologist within 50 miles (hereafter called “local”) of veterans’ VA homesites (per national VA organizational survey data). For the same cohort, we then assessed community-level gynecologist availability; counties with < 2 gynecologists/10,000 women (per the Area Health Resource File) were “inadequate-supply” counties. We examined the proportion of women veterans with local VA gynecologist availability in counties with inadequate versus adequate gynecologist supply, stratified by individual and VA homesite characteristics. Chi-square tests assessed statistical differences.

**Participants:**

All women veteran FY2017 VA primary care users nationally.

**Main Measures:**

Availability of a VA gynecologist within 50 miles of a veteran’s VA homesite; county-level “inadequate-supply” of gynecologists.

**Key Results:**

Among 407,482 women, 9% were in gynecologist supply deserts (i.e., lacking local VA gynecologist and living in an inadequate-supply county). The sub-populations with the highest proportions in gynecologist supply deserts were rural residents (24%), those who got their primary care at non-VAMC satellite clinics (13%), those who got their care at a site without a women’s clinic (13%), and those with American Indian or Alaska Native (12%), or white (12%) race. Among those in inadequate-supply counties, 59.9% had gynecologists at their local VA; however, 40.1% lacked a local VA gynecologist.

**Conclusions:**

Most veterans living in inadequate-supply counties had local VA gynecology care, reflecting VA’s critical role as a safety net provider. However, for those in gynecologist supply deserts, expanded transportation options, modified staffing models, or tele-gynecology hubs may offer solutions to extend VA gynecology capacity.

**Supplementary Information:**

The online version contains supplementary material available at 10.1007/s11606-022-07591-5.

## INTRODUCTION

The Veterans Health Administration (VA) mission of providing comprehensive healthcare for women veterans includes gynecology care, such as advanced procedures not typically available in primary care (colposcopy, endometrial biopsy, hysterectomy, etc.). VA is obligated to provide access to specialty gynecology care for all enrolled women veterans across the country, even those residing in areas with scant healthcare resources^1^ and in rural areas.^2,3^ VA benefits cover gynecology care at VA facilities, or as VA-purchased care via a non-VA gynecologist who is part of VA’s approved community-based provider network.

Geographic access to gynecologists thus relies upon availability of a gynecologist at a woman veteran’s VA facility (or a proximate VA) and/or availability of a gynecologist in the VA’s community network. Historically, there has been geographic variation in VA gynecologist supply.^3,4^ Although the VA has worked to hire more gynecologists, in 2015, 27% of VA healthcare systems lacked an onsite gynecologist.^5^ Thus, use of VA’s community network is fairly common for such services: among women veterans who received care through VA for a gender-specific condition, 24% received gynecology care in the community.^3^ However, not all regions of the country have adequate gynecologist supply^6^, raising the possibility that in some areas, VA may not have a sufficient community-based gynecologist pool to draw upon for their community network.

“Gynecologist supply deserts” would arise in areas of overlapping gaps, i.e., in geographic regions lacking both VA-based and community-based gynecology services. Such deserts have been identified for other types of VA services (e.g., primary care and mental health)^1,7^ but have not been examined for gynecology care. Some subgroups of women (e.g., rural residents, ethnic/racial minorities, veterans getting primary care at VA satellite clinics) may be at particular risk for gaps in access to gynecologic care.^8,9^ In gynecologist supply deserts, gynecologic healthcare needs may go unmet, potentially contributing to preventable morbidity related to missed diagnoses and delayed treatments, and potentially exacerbating disparities.

Our objective was to first characterize the extent to which women veterans live in gynecologist supply deserts (i.e., have both inadequate community gynecologist supply and lack of local VA gynecologists), and second, examine residence in gynecologist supply deserts by individual and VA site characteristics. We also make a novel contribution to the literature that can inform policy and planning, by presenting gynecologist supply deserts geographically.

## METHODS

### Overview

This cross-sectional descriptive study uses VA administrative data and information on county-level clinician supply to characterize veterans with reduced access to gynecology care, either because it is not available locally in the VA, in their community, or both. This work was approved by the VA Central IRB.

### Data

This analysis uses fiscal year 2017 (FY17) data from both VA and publicly available sources. Information about women veterans is drawn from a VA database of patient-level sociodemographic characteristics (age, race/ethnicity, service-connected status, whether the patient is “new” to VA, urban/rural residence). It also indicates the VA site (a VA Medical Center or one of its satellite facilities) where the veteran received care most frequently, or, in the case of a tie, most recently (referred to hereafter as “homesite”). This source database, created by the VA Women’s Health Evaluation Initiative (WHEI) with the support of VA Office of Women’s Health (OWH; VA’s national program office overseeing women’s healthcare delivery nationwide), draws from multiple VA enrollment and utilization files.^2^

Other VA data come from the VA Women’s Assessment Tool for Comprehensive Health (WATCH) survey, which asks site representatives for information about services available at their site. This survey is administered by OWH to each VA site of primary care. In FY17, the WATCH response rate was 100% (*n* = 1197 sites of primary care). This study uses responses about where the site refers women for specialty gynecology care (as opposed to reproductive health services that can be provided in primary care by a non-specialist, which are not explored in this study).

Publicly available data include county-level clinician and population information available from the Health Resources and Services Administration’s Area Health Resource File (AHRF; 2018-2019 release, with data on calendar year 2017).^10^

To create a person-level analysis file, we linked veteran county of residence with county identifiers (5-digit Federal Information Processing System codes used to uniquely identify counties) in the AHRF, and, separately, veteran homesite to site identifiers in WATCH.

### Cohort

The study cohort includes all women veterans nationally with at least one FY17 VA primary care visit (in a general primary care clinic and/or a women’s clinic) (*n* = 417,287). We excluded women with missing data across any of the data sources (*n* = 9805, 2.3%), the majority of whom were missing county codes or individual sociodemographic characteristics. This resulted in an analysis cohort of 407,482 veterans.

### Variables

#### Community Gynecologist Supply

To measure community gynecologist supply, we calculated the number of practicing obstetrician-gynecologists in a county per 10,000 women (veterans and non-veterans) in the county. Using recommended standards for adequate obstetrician-gynecologist availability,^6,11^ we created two categories of county-level supply for main analyses: inadequate (≤ 2 per 10,000 women) and adequate (> 2 per 10,000 women). Women were assigned a level of community gynecologist supply corresponding to their county of residence (per VA Enrollment file data).

#### VA Gynecologist Supply

To measure VA gynecologist supply, we used responses from a WATCH survey question: “Where did women receiving care at this … clinic get specialty gynecology services most often (e.g., for abnormal Pap, abnormal bleeding, gynecology surgery)?” Using the survey response categories determined by the national VA Office of Women’s Health (Web Appendix Table 1), responses for each woman’s homesite were first grouped into categories describing where gynecology services were most often received: (1) at this site, (2) at another VA site within 50 miles, (3) at another VA site beyond 50 miles, and (4) through VA-purchased care at any distance. Based on these categories, we coded VA gynecology services as “Local” if available at the site or within 50 miles, “Distant” if available in VA but beyond 50 miles, and “No VA gynecologist” if only available through VA-purchased care, regardless of the distance.

#### Gynecologist Supply Desert

Women residing in a county characterized as inadequate supply and who lacked a local VA gynecologist are considered to live in a gynecologist supply desert.

#### Patient-Level Characteristics

Sociodemographic variables allowed for comparison of community gynecology care supply with VA gynecology care access within sub-populations of interest: age group, any service-connected disability rating status, rural residence, and race/ethnicity were measured using definitions developed for a series of reports on women veteran VA patients.^2^ We created an additional indicator, “new to VA” status, identifying whether the veteran had a primary care visit in FY17 but no evidence of VA use within the previous 8 years.

#### Homesite-Level Characteristics

We also examined sub-populations based on homesite-level characteristics: whether the homesite was a VA medical center (VAMC) versus another type of VA site (i.e., satellite clinics) and whether it had a separate women’s clinic (i.e., a multidisciplinary clinic offering primary care, mental healthcare, and often other services like gynecology).

### Analyses

We assessed frequencies of sociodemographic and site-level characteristics for the cohort overall, and stratified by residence in a gynecologist supply desert. We also examined the proportion of the cohort residing in a gynecologist supply desert overall, and within each sociodemographic and homesite-level characteristic. We tested whether the proportion of veterans without local VA gynecologists were higher for those in counties with inadequate gynecologist supply compared to those in counties with adequate supply, using a chi-squared test. Conversely, we tested whether the proportion of veterans in inadequate-supply counties was higher for those without local VA gynecologists compared to those with local VA gynecologists.

Finally, we depicted community and VA gynecologist supply data geographically, using four US county maps. The maps dichotomize the VA gynecologist measure to show counties where less than 50% versus 50% or more of the study cohort had a local VA gynecologist, and simultaneously show which counties had inadequate- versus adequate-supply of community gynecologists. Analyses were conducted in SAS® 9.2 (Cary, NC) and maps were created using ArcGIS by Esri (Redlands, CA).

## RESULTS

### Description of Study Cohort

As seen in Table 1, over a quarter of the cohort (26.7%) lived in rural areas, and nearly half were women of color (41.0%). Most were under 65 years old (87.2%), had a service-connected disability rating (67.9%), and/or were returning VA patients (96.5%). Just over half received their primary care at a non-VAMC satellite clinic (53.7%); similarly, just over half received primary care at a site without a women’s clinic (53.6%).

**Table 1:** Distribution of individual and VA homesite characteristics of women veteran VA patients in national study sample, overall, and by gynecologist supply desert status, FY17

			Gynecologist supply desert?^†^	
	Overall (*n* = 407,482)		Yes (*n* = 36,936, 9% of women veterans)	No (*n* = 370,546, 91% of women veterans)
	*N*	%^‡^	%^‡^	%^‡,§^
Individual characteristics				
Rural/urban residence				
Rural	108,789	26.7%	71.3%	22.3%
Urban	298,693	73.3%	28.7%	77.8%
Race/ethnicity				
American Indian or Alaska Native	5,084	1.3%	1.7%	1.2%
White	226,343	55.6%	73.5%	53.8%
Unknown	14,176	3.5%	3.2%	3.5%
Native Hawaiian or other Pacific Islander	4,156	1.0%	0.8%	1.0%
Hispanic	26,442	6.7%	4.3%	7.0%
Black or African American	124,370	30.5%	15.9%	32.0%
Asian	5,911	1.5%	0.6%	1.5%
Age				
18–44 years old	162,096	39.8%	35.1%	40.3%
45–64 years old	193,247	47.4%	49.1%	47.3%
65 years old or older	52,139	12.8%	15.9%	12.5%
Service-connected disability rating status				
Any rating	276,461	67.9%	63.3%	68.3%
None	131,021	32.2%	36.7%	31.7%
New/return status				
New to VA	14,456	3.6%	3.0%	3.6%
Returning to VA	393,026	96.5%	97.0%	96.4%
Characteristics of VA site where woman receives care				
VAMC/other				
VAMC	188,657	46.3%	23.2%	48.6%
Other	218,825	53.7%	76.8%	51.4%
Women’s clinic at site				
Women’s clinic at site	189,267	46.5%	20.5%	49.0%
No women’s clinic at site	218,215	53.6%	79.5%	51.0%

*VA*, Veterans Health Administration; *FY*, fiscal year; *VAMC*, Veterans Administration Medical Center; *Other*, community-based outpatient clinic or other non-VAMC VA site

†Women residing in a gynecologist supply desert have both inadequate community supply in the county of residence (2 or fewer gynecologists per 10,000 women) and no local VA gynecologist (i.e., no VA gynecologist was available at the homesite or within 50 miles of the homesite)

‡Percents use the column total as the denominator. For example, among women in a gynecologist supply desert, 71.3% had rural residences

^§^Distributions of all characteristics reported in Table 1 among those who do not reside in a gynecologist supply desert (*n* = 370,546) are significantly different from the sub-population who reside in a gynecologist supply desert (*n* = 36,936) (*p* < 0.001 for each characteristic)

^¶^Sites reference veterans’ homesites

### Gynecologist Supply Deserts, Overall and by Sub-population

Overall, 9% (*n* = 36,936 women) of the study cohort lived in gynecologist supply deserts, both lacking a local VA gynecologist and living in an inadequate-supply county (Table 1). Among them, 56% (*n* = 20,622 women) did not have a distant VA gynecologist (i.e., they would need to rely on a community provider for gynecology care) (data not shown).

**Figure 1:**
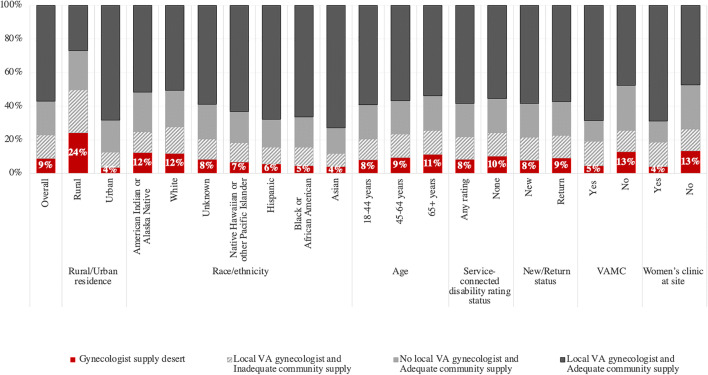
Percent of veterans in gynecologist supply deserts, across individual and homesite characteristics, FY17. VA, Veterans Health Administration. Notes: (1) Women residing in a gynecologist supply desert have both inadequate community supply in the county of residence (2 or fewer gynecologists per 10,000 women) and no local VA gynecologist (i.e., a gynecologist was available at the homesite or within 50 miles of the homesite, but still in VA). (2) Percents in figure use the total number of women with characteristic (as reported in column of Table 1) as the denominator. For example, among all rural women veterans (*n* = 108,789 women), 24% reside in a gynecologist supply desert. (3) “VAMC” and “women’s clinic at site” refers to the veterans’ homesite (i.e., where they get most of their primary care).

The proportion of the women in each sub-population who lived in a gynecologist supply desert varied (4–24%), as shown in Figure 1. The sub-populations with the highest proportions were rural residents (24%), those who got their primary care at non-VAMC satellite clinics (13%), those who got their care at a site without a women’s clinic (13%), and those with American Indian or Alaska Native (12%), or white (12%) race.

### Comparison of VA Gynecologist to Community Gynecologist Supply

Overall, most veterans (70.8%) had a local VA gynecologist, but a substantial group had either a distant (11.7%) or no VA gynecologist (17.5%) (data not shown). Veterans without a local VA gynecologist were more likely to live in inadequate-supply counties and vice versa. The percent of veterans without a local VA gynecologist was higher among veterans living in an inadequate-supply (versus adequate-supply) county (40.1% versus 26.0%) (*p* < 0.001). Conversely, the percent of veterans who lived in an inadequate-supply county was higher among veterans with distant or no VA gynecologist (versus local VA gynecologist) (34.3%, 28.9% versus 19.1%) (data not shown).

### Geographic Distribution of Gynecologist Supply Deserts

**Figure 2:**
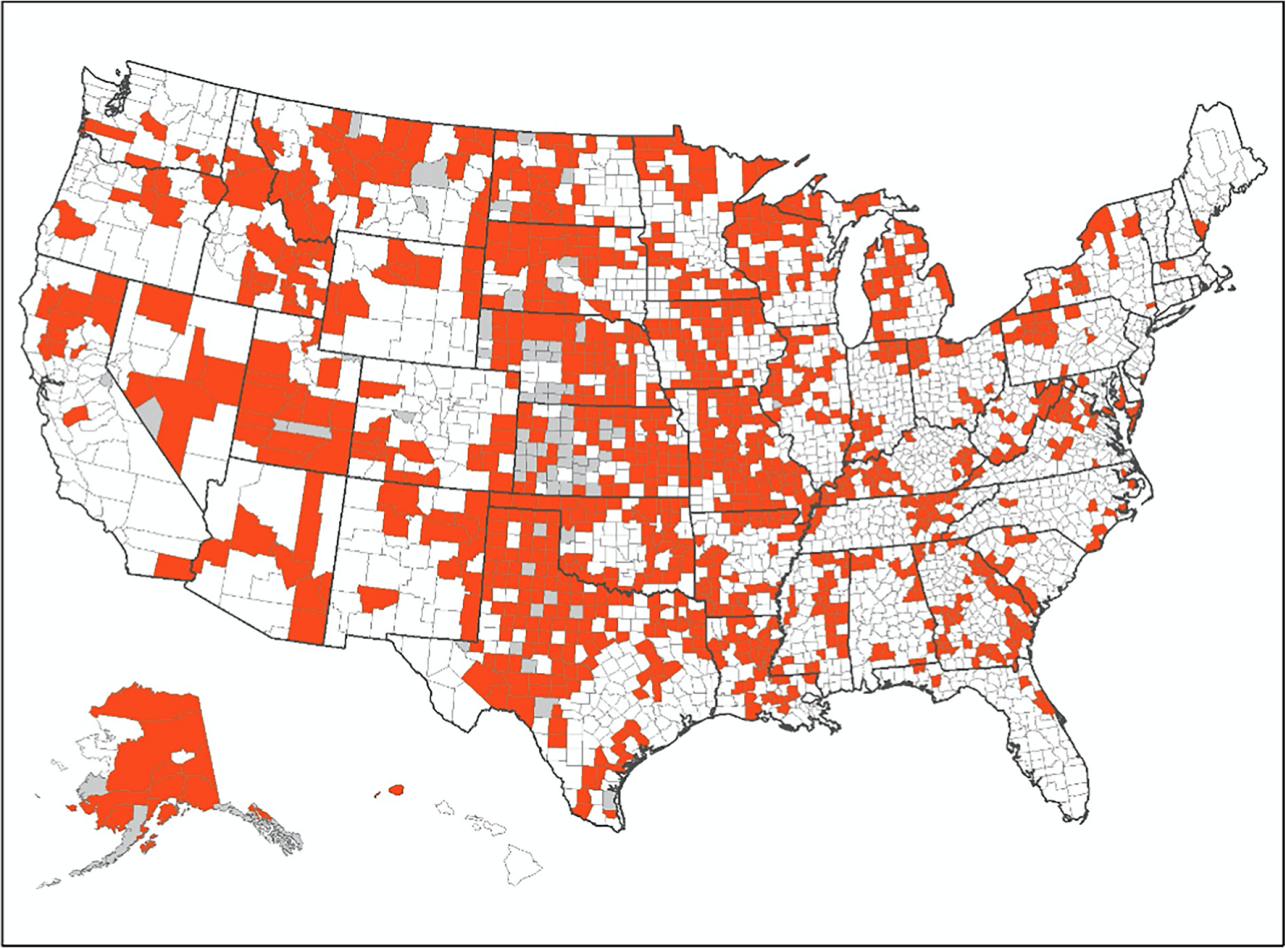
US counties characterized as gynecologist supply deserts (*n* = 1130 counties, 37% of counties). Notes: (1) For this figure, counties are the unit of analysis. (2) Map indicates counties characterized as gynecologist supply deserts. These counties have 2 or fewer gynecologists per 10,000 women and at least half of women veteran VA primary care patients had a VA homesite without a local VA gynecologist. Having a local VA gynecologist means that there were gynecology services at the woman’s VA homesite or at a VA within 50 miles of the  homesite.

The map in Figure 2 shows counties that could be characterized as gynecologist supply deserts, as they had inadequate supply of gynecologists and the majority of women veterans living in the county lacked a local VA gynecologist. There were 1130 counties (37% of all counties) meeting these criteria. They were located primarily in the Midwest and mountain west regions. Web Appendix 4 includes maps of counties that are not gynecologist supply deserts, either due to their VA gynecologist supply only (*n* = 816 counties), their community gynecologist supply only (*n* = 534 counties), or both VA and community gynecologist supply (*n* = 579 counties).

## DISCUSSION

We found that nearly 1 in 10 women veteran VA primary care patients lived in a gynecologist supply desert in 2017, with no local VA gynecologist and with inadequate county-level gynecologist supply. Subgroups at particular risk of residing in a gynecologist supply desert included those living in rural areas, women veterans with American Indian/Alaska Native or white race, as well as those receiving primary care at satellite clinics and those receiving primary care at sites without a women’s clinic. VA policy entitles veterans lacking timely or nearby VA care to obtain care from community clinicians.^12^ However, this policy alone may not suffice for ensuring access in areas that also have scarcity of community gynecologists.

We identify nearly 37,000 women veterans who may face barriers to accessing gynecology services because they live in counties with inadequate gynecologist supply and also lack a local VA gynecologist. For them, greater use of VA-purchased care in the community may not help improve access, since their local communities also have insufficient gynecologist supply. These women may therefore lack timely access to reproductive healthcare when the need arises, which, in turn, may have detrimental health effects. Notably, high proportions of American Indian/Alaska Native women live in such gynecologist supply deserts. While some American Indian/Alaska Native women may have access to care through the Indian Health Service, limitations to gynecology care access for this group is a concern, and may exacerbate known health inequities and well-documented disparities in all-cause mortality.^13^

That women in inadequate-supply counties more commonly lack a local VA gynecologist is concerning but not surprising. From the institutional administrator perspective, some VA facilities in low-supply counties may see a low volume of women veterans, making it fiscally challenging to recruit and maintain an onsite gynecologist.^5^ From the physician perspective, the same factors that drive health workforce shortages in rural and other under-served areas^14,15^ may also make physicians less inclined to be recruited to a VA facility in those areas. These may include limited job opportunities for spouses,^16^ lack of familiarity with rural lifestyles,^17^ and less enticing financial incentives.^18^ Workforce recruitment challenges in VA merit further inquiry.

Given well-documented clinician shortages in rural areas,^14^ it may not be surprising that rural veterans, regardless of community supply, frequently lacked local VA gynecologists. More surprisingly however, over a quarter (28%) of *urban* veterans in low-supply areas also lacked a local VA gynecologist. While small urban areas (which may face issues similar to those faced in rural areas) count as urban, these findings suggest that future research on access to care in the VA should consider area clinician supply in addition to rural/urban status.

While many women lived in gynecology deserts, it is notable that over half of the veterans residing in inadequate-supply counties *did* have local VA gynecology care: without VA, these women would likely have few alternative sources for gynecology care. This highlights the important role VA plays as a safety net provider in medically under-resourced areas.^19,20^ Public funding for the VA allows it to maintain access points in areas less able to attract or sustain private healthcare providers, thereby creating vital healthcare infrastructure. In these areas, women’s ability to receive needed gynecology specialty services may benefit from VA policy to maximize outreach to women veterans who do not use VA^2^ and non-VA policy to address access to gynecologists for non-Veteran women who do not have access to VA infrastructure.

This study echoes related analyses of medical deserts across VA and community providers. For example, nearly a quarter of veterans enrolled in the VA live in a county that was both a healthcare shortage area (as defined by the Health Resources and Services Administration) and did not have a VA site of care.^1^ Similarly, Ohl and colleagues point to high proportions of veterans who are eligible for VA-purchased care (by virtue of their proximity to the nearest VA site), who also live in healthcare shortage areas.^7^ The present study expands this inquiry through a focus on gynecology care in a national cohort of women veterans.

Limitations to the community gynecologist supply measure include (1) this measure does not account for the fact that non-gynecologists (e.g., family physicians, nurse practitioners) sometimes provide at least limited gynecology services;^21^ (2) not all obstetrician-gynecologists counted in the community gynecologist measure offer the full spectrum of gynecology services, suggesting that service gaps could exist even where a gynecologist is available; (3) not all community gynecologists are part of the VA-purchased care networks, and those that are may not have appointment availability. Limitations to the local VA gynecologist measure include (1) its reliance on self-reported information from VA sites of care (introducing potential measurement error); (2) lack of VA gynecologist supply adjustment per-capita (i.e., to account for variation in number of women veterans served per VA site); (3) the threshold distance (50 miles) used to define “local” VA gynecologist may exceed that distance to her residence.

## CONCLUSIONS

Study findings have two parallel policy implications for VA. First, in gynecologist supply deserts, relying solely on VA-purchased care may not suffice to alleviate access issues. In these areas, attention to hiring VA gynecologists, extending service capacity via non-gynecologist clinicians (e.g., family medicine physicians, nurse practitioners) who have specialty gynecology skills, expansion of veteran transportation options to specialty gynecology locations, and innovation around staffing models (e.g., VA-based traveling clinicians, or tele-gynecology hubs) may offer solutions for local VAs with low volumes of women patients. A VA demonstration project of a provider-to-provider women’s health educational and virtual consultation program found the virtual format to be a promising modality for positively influencing patient care.^22^ That demonstration project subsequently demonstrated the feasibility of providing tele-gynecology consultations with that format. National organizations that provide widely accessible women’s healthcare via models in which physicians lead teams of advance practice providers (e.g., nurse practitioners and nurse midwives) could also serve as a model for VA in ensuring access to cost-effective gynecological services in rural areas.

Second, in areas where lack of VA gynecologists correlates with greater gynecologist supply in the surrounding community, it is important that community-based gynecologists are included in the contracted networks used for VA-purchased care referrals,^23^ that VA monitors the quality of these community-based providers’ care, and that robust systems for care coordination between VA and non-VA clinicians are in place.^24^ Ideally such community-based gynecologists would be versed in distinct characteristics of women veterans, such as the high rates of military sexual trauma and PTSD in this population,^2^ which may necessitate coordination with VA-based mental health providers^24^ and attention to trauma-informed care.^25^ It is also important for women veterans to know they can identify in-network gynecologists by selecting “community providers (in VA’s network)” on the VA facility locator,^26^ with the caveat that the locator does not indicate whether a specific clinician is accepting new patients. Additionally, veterans may have options for a purchased-care clinician versus gynecologist at a distant VA site when a gynecologist is not available at a local VA, though more research is needed to understand how veterans experience this choice.

As the number of women veterans in VA has grown,^2^ access to gynecology care has become even more salient. This study identified a large cohort of veterans in gynecologist supply deserts, who likely had scarce access to gynecology care both within and outside of VA. Remaining true to VA’s mission to care for all veterans, regardless of gender and no matter how remote, will require continued attention to approaches that overcome gaps in gynecologist supply.

## Supplementary Information

ESM 1 (DOCX 789 kb)
