# Supplementary material for: Gynecologist Supply Deserts Across the VA and in the Community
Source: J Gen Intern Med. 2022 Aug 30;37(Suppl 3):690–7. doi: 10.1007/s11606-022-07591-5 (PMC9481821; doi:10.1007/s11606-022-07591-5)
Supplement: Supplementary file 1 — (DOCX 789 kb) [file 11606_2022_7591_MOESM1_ESM.docx]

**WEB APPENDICES**

| Web Appendix Table 1. Categorizing WATCH FY17 response values into VA gynecology care variable values (grouping 2) | | |
| --- | --- | --- |
| WATCH FY17 value† | Grouping 1: Where VA clinic refers for gynecology care | Grouping 2: VA gynecology care? |
| 1. At this clinic | At this VA site | Local |
| 2. At another clinic within this VA Site |  |  |
| 3. At another site within this HCS: within 50 miles | At another VA site within 50 miles |  |
| 5. At another VA outside this HCS: within 50 miles |  |  |
| 4. At another site within this HCS: more than 50 miles away | At another VA site beyond 50 miles | Distant only |
| 6. At another VA outside this HCS: more than 50 miles away |  |  |
| 7. Through non-VA (fee) care in the community of Choice/contract services: within 50 miles | VA community care | None |
| 8. Through non-VA (fee) care in the community or Choice/contract services: more than 50 miles |  |  |
| 9. Other | Missing data | Missing data |

†Includes values from three separate gynecology variables in the WATCH data, each corresponding to a different model of primary care.

Web Appendix Table 2. Distribution of individual and VA homesite characteristics of study sample, by VA and community gynecology supply, FY17

|  | Gynecology supply desert† (n=36,936) | No local VA gynecologist /Adequate-supply county (n=81,997) | Local VA gynecologist /Inadequate-supply county (n=55,198) | Local VA gynecologist /Adequate-supply county  (n=233,351) |
| --- | --- | --- | --- | --- |
| **Individual characteristics** | %^‡^ | %^‡^ | %^‡^ | %^‡^ |
| **Rural/Urban residence** |  |  |  |  |
| Rural | 71.3% | 31.1% | 50.1% | 12.6% |
| Urban | 28.7% | 68.9% | 49.9% | 87.4% |
| **Race/ethnicity** |  |  |  |  |
| American Indian or Alaska Native | 1.7% | 1.5% | 1.2% | 1.1% |
| White | 73.5% | 59.7% | 64.9% | 49.1% |
| Unknown | 3.2% | 3.6% | 3.1% | 3.6% |
| Native Hawaiian or other Pacific Islander | 0.8% | 1.0% | 0.9% | 1.1% |
| Hispanic | 4.3% | 5.6% | 4.9% | 8.0% |
| Black or African American | 15.9% | 27.6% | 24.4% | 35.3% |
| Asian | 0.6% | 1.1% | 0.8% | 1.8% |
| **Age** |  |  |  |  |
| 18-44 years old | 35.1% | 40.2% | 36.9% | 41.1% |
| 45-64 years old | 49.1% | 46.7% | 49.5% | 46.9% |
| 65 years old or older | 15.9% | 13.1% | 13.6% | 12.0% |
| **Service-connected disability rating status** |  |  |  |  |
| Any rating | 63.3% | 67.1% | 67.3% | 69.0% |
| None | 36.7% | 32.9% | 32.7% | 31.1% |
| **New/Return status** |  |  |  |  |
| New to VA | 3.0% | 3.5% | 3.6% | 3.6% |
| Return VA | 97.0% | 96.5% | 96.4% | 96.4% |
| **Characteristics of VA site where woman receives care**^§^ |  |  |  |  |
| **VAMC/Other** |  |  |  |  |
| VAMC | 23.2% | 28.5% | 49.9% | 55.4% |
| Other | 76.8% | 71.5% | 50.1% | 44.7% |
| **Women’s clinic** |  |  |  |  |
| Women’s clinic | 20.5% | 30.3% | 48.8% | 55.7% |
| No women’s clinic | 79.5% | 69.7% | 51.2% | 44.3% |

VA: Veterans Health Administration; FY: Fiscal Year; VAMC: Veterans Administration Medical Center; Other: Community-based outpatient clinic or other non-VAMC VA site

†Women residing in a gynecologist supply desert have both inadequate community supply in the county of residence (2 or fewer gynecologists per 10,000 women) and no local VA gynecologist (i.e., no VA gynecologist was available at the homesite or within 50 miles of the homesite).

‡Percents use the column total as the denominator. For example, among women in a gynecologist supply desert, 71.3% had rural residences.

^§^Sites reference veterans’ homesites.

Web Appendix Table 3. Percent of veterans by VA and community gynecology supply, across individual and homesite characteristics, FY17

|  | Gynecologist supply desert† (9%) | No local VA gynecologist /Adequate-supply county (20%) | Local VA gynecologist /Inadequate-supply county (14%) | Local VA gynecologist /Adequate-supply county (57%) |
| --- | --- | --- | --- | --- |
|  | Row % | | | |
| **Individual characteristics** |  |  |  |  |
| **Rural/Urban residence** |  |  |  |  |
| Rural | 24.2% | 23.4% | 25.4% | 27.0% |
| Urban | 3.6% | 18.9% | 9.2% | 68.3% |
| **Race/ethnicity** |  |  |  |  |
| American Indian or Alaska Native | 12.3% | 23.6% | 12.5% | 51.6% |
| White | 12.0% | 21.6% | 15.8% | 50.6% |
| Unknown | 8.4% | 20.6% | 12.0% | 59.0% |
| Native Hawaiian or other Pacific Islander | 6.8% | 18.7% | 11.4% | 63.0% |
| Hispanic | 5.7% | 16.9% | 9.8% | 67.6% |
| Black or African American | 4.7% | 18.2% | 10.8% | 66.3% |
| Asian | 4.0% | 15.4% | 7.9% | 72.8% |
| **Age** |  |  |  |  |
| 18-44 years old | 8.0% | 20.3% | 12.6% | 59.1% |
| 45-64 years old | 9.4% | 19.8% | 14.1% | 56.7% |
| 65 years old or older | 11.2% | 20.5% | 14.4% | 53.8% |
| **Service-connected disability rating status** |  |  |  |  |
| Any rating | 10.4% | 19.9% | 13.4% | 58.2% |
| None | 8.5% | 20.6% | 13.8% | 55.3% |
| **New/Return status** |  |  |  |  |
| New to VA | 9.1% | 20.0% | 13.9% | 58.4% |
| Returning to VA | 7.8% | 20.1% | 13.5% | 57.2% |
| **Characteristics of VA site where woman receives care**^§^ |  |  |  |  |
| **VAMC/Other** |  |  |  |  |
| VAMC | 4.5% | 12.4% | 14.6% | 68.5% |
| Other | 13.0% | 26.8% | 12.6% | 47.6% |
| **Women’s clinic** |  |  |  |  |
| Women’s clinic at site | 4.0% | 13.1% | 14.2% | 68.6% |
| No women’s clinic at site | 13.5% | 26.2% | 13.0% | 47.4% |

VA: Veterans Health Administration; FY: Fiscal Year; CBOC: Community-based outpatient clinic; VAMC: Veterans Administration Medical Center

†Women residing in a gynecologist supply desert have both inadequate community supply in the county of residence (2 or fewer gynecologists per 10,000 women) and no local VA gynecologist (i.e., a gynecologist was available at the homesite or within 50 miles of the homesite, but still in VA).

‡Percents use the row total as the denominator. For example, among rural women, 24% lived in a gynecologist supply desert. ^§^Sites reference veterans’ homesites.

Web Appendix Figure 4. U.S. Counties by community and VA gynecologist supply


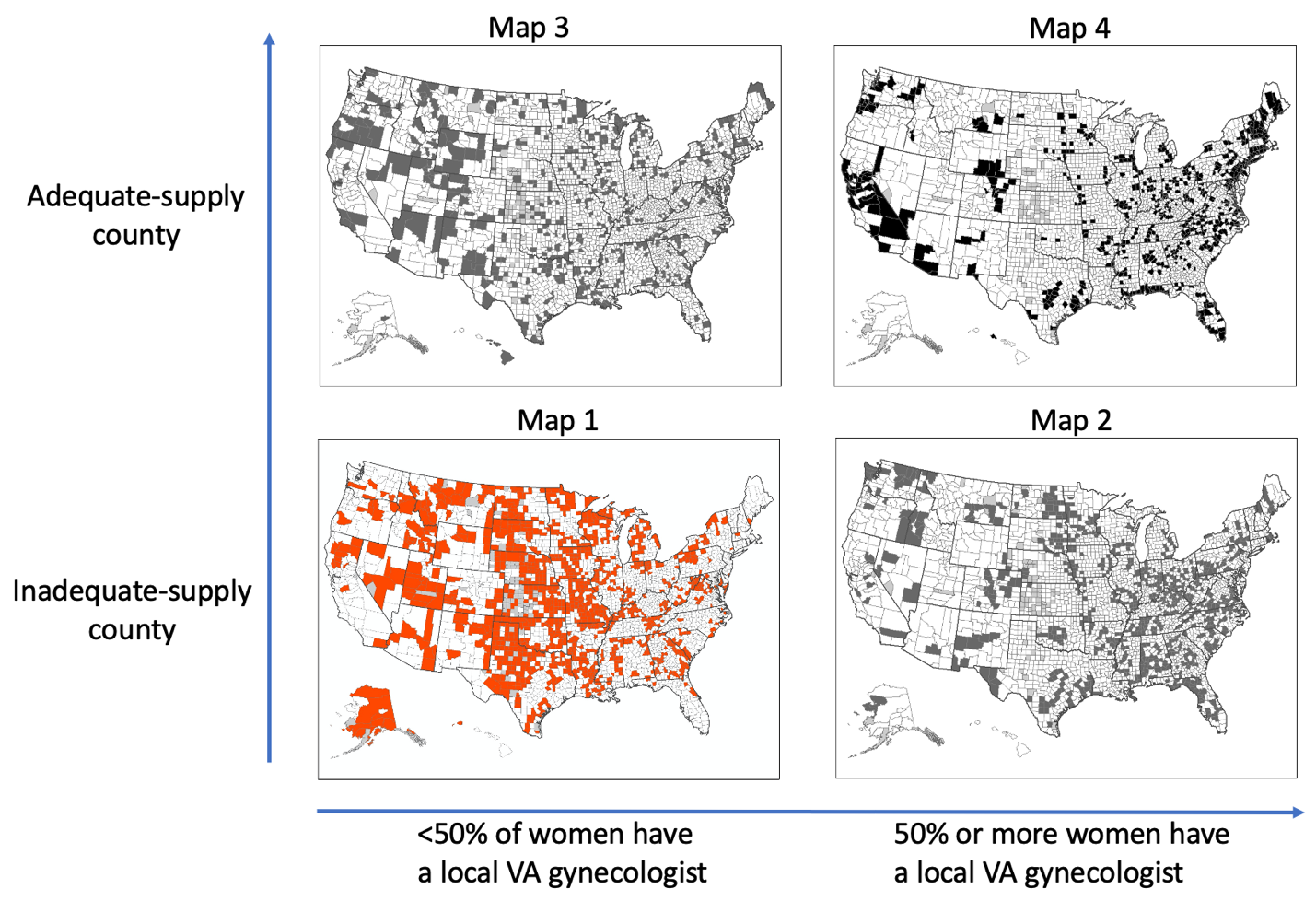


VA: Veterans Health Administration

Notes: 1. For this figure, counties are the unit of analysis. 2. Community gynecologist supply measures the total number of practicing gynecologists per 10,000 women in a county. For the y-axis, inadequate-supply counties have 2 or fewer gynecologists per 10,000 women, and adequate-supply counties have more than 2 gynecologists per 10,000 women. Counties are based on veteran’s county of residence. 3. For the x-axis, counties are dichotomized into those where half of women veteran VA primary care patients had a VA homesite with a local VA gynecologist. Having a local VA gynecologist means that there were gynecology services at the woman’s VA homesite or at a VA within 50 miles of her homesite. 4. Light grey refers to counties where none of the study cohort resided. Map 1: n=1,130 counties (37% of counties); Map 2: n=816 counties (27% of counties); Map 3: n=534 counties (17% of counties); Map 4: n=579 counties (19% of counties).
